# Efficacy of Age-Adjusted Dyspnea, Eosinopenia, Consolidation, Acidemia and Atrial Fibrillation Score in Predicting Long-Term Survival in COPD-Related Persistent Hypercapnic Respiratory Failure

**DOI:** 10.3390/life15040533

**Published:** 2025-03-24

**Authors:** Maşide Ari, Emrah Ari

**Affiliations:** 1 Department of Pulmonology, Ankara Atatürk Sanatorium Training and Research Hospital, 06290 Ankara, Turkey; 2 Department of Emergency Medicine, Mamak Public Hospital, 06270 Ankara, Turkey; dremrahari25@gmail.com

**Keywords:** A-DECAF, DECAF, HRF, survival

## Abstract

**Background and Objectives**: Hypercapnic respiratory failure (HRF) is a critical clinical condition commonly encountered in acute exacerbations of chronic obstructive pulmonary disease (COPD), leading to high morbidity and mortality rates. The existing scoring systems have primarily been developed for short-term mortality prediction, and their impact on long-term survival has not been sufficiently investigated. This study aims to identify the prognostic factors affecting long-term survival in patients with persistent HRF due to COPD and to evaluate the effectiveness of the Age-Adjusted DECAF (A-DECAF) score, which was created by incorporating the age variable into the existing DECAF score, in predicting long-term survival. **Materials and Methods**: This retrospective study included patients admitted to an intensive care unit from an emergency department with HRF between April 2022 and November 2023. The demographic data, comorbidities, the laboratory results, and the treatment protocols were recorded. The A-DECAF scores were calculated and analyzed using Kaplan–Meier and ROC analyses. Survival assessment was conducted with Kaplan–Meier analysis, while univariate and multivariate Cox regression analyses were performed to identify the prognostic factors. **Results**: Among 357 patients, 24.4% died within one year after discharge. The deceased patients were significantly older (*p* < 0.001) and had higher APACHE-II, DECAF, and A-DECAF scores (*p* < 0.001 for all). ROC analysis showed that the A-DECAF score had the highest sensitivity (93.1%) and accuracy (AUC = 0.813) for survival prediction. Kaplan–Meier analysis indicated lower survival rates with increasing A-DECAF scores. Cox regression identified the A-DECAF score as the strongest independent predictor (*p* < 0.001), while lung cancer (*p* = 0.044) and invasive mechanical ventilation (*p* = 0.039) also negatively impacted survival. **Conclusions**: The A-DECAF score is an effective tool for predicting long-term survival in patients with COPD and persistent HRF, particularly aiding clinical decisions regarding elderly populations. Further research is needed to validate its use in diverse patient groups.

## 1. Introduction

Hypercapnic respiratory failure (HRF) is a type of respiratory failure characterized by the increase in arterial carbon dioxide pressure (pCO_2_ > 45 mmHg) [1]. HRF developing during acute exacerbations of chronic obstructive pulmonary disease (COPD) is a significant clinical condition associated with severe morbidity and mortality [2]. HRF frequently necessitates invasive or non-invasive mechanical ventilation support, and as a result emerges as a significant cause of intensive care unit (ICU) admissions [3]. Although numerous studies in the available literature focus on HRF-related mortality and the effects of non-invasive mechanical ventilation, the data on the long-term prognosis of these patients remain limited. In particular, the fact that the existing scoring systems are typically designed to evaluate short-term outcomes creates a significant gap in this area. There is a critical need to develop new scoring systems that include factors such as age, comorbidity burden, and their impact on long-term survival to enhance clinical decision-making processes.

In this context, the DECAF (Dyspnea, Eosinopenia, Consolidation, Acidemia and Atrial Fibrillation) score, which is widely used for mortality prediction in acute exacerbations of COPD (AE-COPD), has been shown to have high sensitivity and specificity in predicting in-hospital short-term mortality [4]. However, its effectiveness in predicting long-term prognosis has not been sufficiently investigated. The DECAF score does not include age, an important prognostic factor, which poses significant limitations in estimating long-term survival, particularly in elderly patient populations. Many studies have emphasized that age is not only a risk factor for mortality, but also a key determinant of long-term prognosis [5]. The Age-Adjusted DECAF (A-DECAF) score was developed to improve the accuracy of survival predictions by incorporating age as a variable. It was designed to evaluate long-term survival predictions in patients who develop persistent HRF due to COPD.

## 2. Materials and Methods

This study was conducted between April 2022 and November 2023 in the respiratory intensive care unit of our hospital among patients monitored for HRF. The patients were retrospectively evaluated based on predefined criteria during their ICU stay and were subsequently followed prospectively for long-term survival. Therefore, our study was designed as a retro-prospective research model, incorporating both retrospective and prospective components.

This study was initiated with the approval of the Clinical Research Ethics Committee of Ankara Atatürk Sanatorium Training and Research Hospital (approval number 187, dated 25 December 2024) and was conducted in accordance with the ethical principles outlined in the Declaration of Helsinki.

Our study was conducted at a tertiary care training and research hospital, which serves as a reference center in the field of respiratory medicine. Our hospital houses both secondary- and tertiary-level respiratory intensive care units, with a high number of patients being monitored for HRF. Therefore, the study center possesses extensive clinical experience in this area, enabling the comprehensive evaluation of patient care processes.

The medical treatment of the patients was planned based on national and international guidelines. Invasive or non-invasive mechanical ventilation methods were utilized for the treatment of HRF. Non-invasive mechanical ventilation (NIMV) was mostly applied using an oronasal mask. Tidal volumes were calculated based on the patients’ estimated ideal body weight. The patients were continuously monitored, and oxygen support was adjusted to maintain arterial oxygen saturation within the range of 90–92%. The response to treatment was evaluated based on vital signs, such as respiratory rate, blood pressure, and heart rate, as well as arterial blood gas (ABG) parameters. The arterial blood gas results obtained at the time of ICU admission and prior to discharge were also recorded.

All the patients included in our study had been previously diagnosed with COPD by a respiratory physician using pulmonary function testing (PFT). The further analysis of their GOLD stages revealed that the majority of the patients were classified as GOLD stage 3 or 4.

As part of this study, the demographic and clinical characteristics of the patients, such as age, gender, comorbidities, and medications, were thoroughly evaluated. Additionally, the blood tests results conducted within the first 24 h of hospital admission, the ABG results obtained at discharge, and the applied treatment protocols were examined. The primary outcome of this study was defined as the time from the date of HRF diagnosis to the date of death. The relationship between the obtained parameters and one-year post-discharge survival was comprehensively analyzed.

### 2.1. Determination of Long-Term NIMV Requirement and Persistent Hypercapnic Respiratory Failure

In our intensive care unit, the need for NIMV is assessed and managed according to specific criteria. For patients experiencing HRF for the first time, NIMV support was initiated, and subsequently weaned in a stepwise manner based on the following criteria.

After the patient’s vital signs stabilized and the acute condition causing hypercapnic respiratory failure resolved, the ABG parameters were monitored. When the ABG values improved (pH ≥ 7.35, pCO_2_ ≤ 55 mmHg), NIMV support was gradually reduced. During the weaning process, daytime NIMV support was discontinued first, followed by a stepwise reduction in night-time support. Once the ABG parameters reached pH ≥ 7.35 and pCO_2_ ≤ 50 mmHg, NIMV was completely withdrawn.

Following NIMV discontinuation, the patient’s ABG values and vital signs were regularly monitored. If during the weaning process there was a more than 20% increase in PaCO_2_ compared to the baseline, an increase in respiratory rate, and the development of severe dyspnea with pH ≤ 7.35, NIMV support was reinstated. This condition was considered as persistent hypercapnia. These patients were defined as the group requiring long-term NIMV and were included in this study.

### 2.2. Diagnosis of Emphysema

In our study, the diagnosis of emphysema was based on high-resolution computed tomography (HRCT) findings. The HRCT evaluation criteria for emphysema included parenchymal destruction, airway enlargement, and the presence of low-density areas. Thoracic CT scans performed either during hospitalization or prior to admission were reviewed by pulmonology and radiology specialists to confirm the presence of emphysema.

### 2.3. Patient Selection

**Figure 1:**
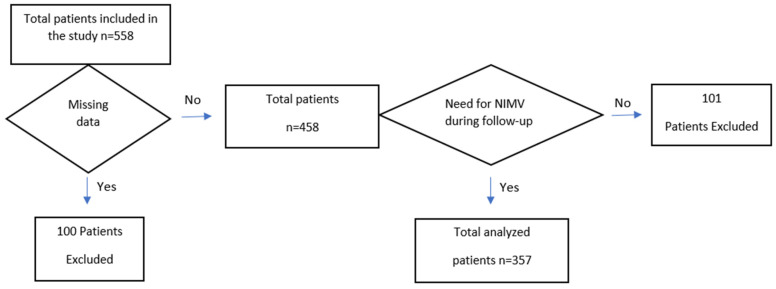
Flowchart of patients included in and excluded from this study.

This study included patients who developed HRF for the first time due to acute exacerbation of COPD (AECOPD) and were subsequently monitored in the intensive care unit. Patients meeting the inclusion criteria and not presenting any of the exclusion criteria were enrolled in this study. Figure 1 shows a flowchart detailing the patients included in and excluded from this study.

#### 2.3.1. Inclusion Criteria

- Patients diagnosed with HRF for the first time due to AE-COPD;
- Patients diagnosed with HRF due to AE-COPD in the emergency department, and subsequently monitored in the intensive care unit;
- Patients with a confirmed diagnosis of COPD through PFT prior to hospital admission;
- Patients who underwent ABG analysis at the time of presentation;
- Patients requiring continued NIMV support during follow-up and in the long term.

#### 2.3.2. Exclusion Criteria

- Patients who did not consent to participate in this study;
- Patients without a confirmed diagnosis of COPD prior to this study;
  ○ Patients with HRF caused by procedures requiring general anesthesia and/or sedation;
- Patients whose HRF was suspected to be caused by opioid use;
- Patients with HRF attributed to cardiac causes;
- Patients with HRF resulting from interstitial lung disease;
- Patients with HRF due to neuromuscular disease;
- Patients with HRF related to obstructive sleep apnea syndrome;
- Patients whose need for NIMV resolved during follow-up and in the long term;
- Patients with a length of stay less than one day;
- Patients with unavailable PFT results prior to hospital admission;
- Patients with missing ABG analysis data at presentation (including those whose initial blood gas in the emergency department was venous, preventing the accurate assessment of acidemia);
- Patients with missing modified Medical Research Council (mMRC) dyspnea scale data (excluded due to the inability to assess dyspnea severity if mMRC level was not clearly documented in medical records);
- Patients with prior glucocorticoid use (excluded due to the potential suppression of eosinophil levels in those who had been using systemic glucocorticoids before hospital admission).

### 2.4. Determination of Follow-Up Period and Long-Term Survival

In our study, long-term survival was defined as the follow-up of patients who survived initial hospitalization, and we tracked them after discharge until death occurred or for a maximum duration of one year (until 1 December 2024). Accordingly, the follow-up period for the primary outcome was defined as the time from the date of HRF diagnosis to the date of death. The participants were followed from their initial admission until death occurred or for a maximum duration of one year (until 1 December 2024). Therefore, the patients who died within the one-year follow-up period were not excluded from analysis, as the entire patient population was considered when evaluating survival time.

### 2.5. Calculation of the DECAF Score

This score is based on the assessment of the five key clinical parameters, each represented by a specific point value. An appropriate score is assigned for the presence of each parameter.

1. **Dyspnea:** Scored based on the British Medical Research Council (mMRC) dyspnea scale.
2. **Eosinopenia:** A score is added if the blood eosinophil level is below 0.05 × 10^9^/L.
3. **Presence of Consolidation:** A score is added in the presence of radiological consolidation.
4. **Acidosis:** Blood gas analysis pH < 7.30 is considered a high-risk factor, so a score is added.
5. **Atrial Fibrillation:** The presence of atrial fibrillation is considered an additional risk factor, so a score is added.

The DECAF score was calculated based on the patient’s initial clinical assessment, laboratory findings, and radiological evaluations performed at the time of emergency department admission. The scores from each parameter were summed to calculate the total DECAF score. Based on the total score, the patients were categorized into the following risk groups:

- **Low risk:** 0–1 points;
- **Moderate risk:** 2 points;
- **High risk:** ≥3 points.

### 2.6. Calculation of the A-DECAF Score

The A-DECAF score was developed by incorporating age as a factor into the original DECAF score. While the five core parameters of the DECAF score were preserved, age was added as an additional variable. In this modified system, 1 point is added to the score if the patient’s age is ≥65 years. With the integration of the age factor, the total A-DECAF scores range from 0 to 7.

### 2.7. Statistical Analysis

The data collected in this study were analyzed using appropriate statistical methods to evaluate the factors affecting survival. The normality of continuous variables was assessed using the Kolmogorov–Smirnov test. For variables with a normal distribution, the results are presented as mean ± standard deviation (mean ± SD), whereas for non-normally distributed variables, they are expressed as median (interquartile range). Categorical variables are reported as frequency and percentage (%).

For comparisons between groups, the Independent Sample *t*-test or Mann–Whitney U test was used for continuous variables, while the Chi-square test or Fisher’s Exact Test was applied for categorical variables. Survival analyses were conducted using the Kaplan–Meier method, and differences between groups were evaluated with the log-rank test. Univariate and multivariate Cox regression analyses were performed to identify the prognostic factors associated with survival, and hazard ratios (HR) with 95% confidence intervals (95% CIs) were calculated. To assess the performance of the APACHE-II, DECAF, and A-DECAF scores in predicting survival, Receiver Operating Characteristic (ROC) analysis was performed. Analysis provided values for the Area Under the Curve (AUC), cut-off points, sensitivity, specificity, positive predictive value (PPV), negative predictive value (NPV), and positive/negative likelihood ratios (LR+/LR−). A significance level of *p* < 0.05 was considered for all analyses, and statistical evaluations were performed using SPSS version 27.0.

## 3. Results

Initially, 558 patients were included in this study. However, 100 patients were excluded due to incomplete data (e.g., missing ABG analysis data at hospital admission, PFT results, or key clinical parameters required for DECAF score calculation, all of which are critical for accurate risk stratification). Additionally, 101 patients who no longer required NIMV during the follow-up period were also excluded. Consequently, analyses were conducted on 357 patients. It was observed that 24.4% of the patients died within one year after discharge.

All the patients included in this study had a confirmed diagnosis of COPD. Upon evaluating them for other respiratory comorbidities, the number of patients diagnosed with emphysema was 116 (32.5%), and there were 3 with asthma–COPD overlap (0.8%), 22 with lung cancer (6.2%), and 22 with a history of pulmonary embolism (6.2%).

The results of comparative analysis based on the survival status of the patients are presented in Table 1. The mean age of the deceased patients was statistically significantly higher compared to that of the surviving patients (*p* < 0.001). When evaluated in terms of the scoring systems, the APACHE-II, DECAF, and A-DECAF scores of the deceased patients were also found to be significantly higher (*p* < 0.001; *p* < 0.001; *p* < 0.001).

The comparison of the laboratory findings from blood tests based on the survival status of the patients is presented in Table 2. According to arterial blood gas analysis, the pH levels were found to be lower in the deceased patients (*p* < 0.001), while the pCO_2_ levels were statistically significantly higher in the deceased patients (*p* = 0.003).

**Figure 2:**
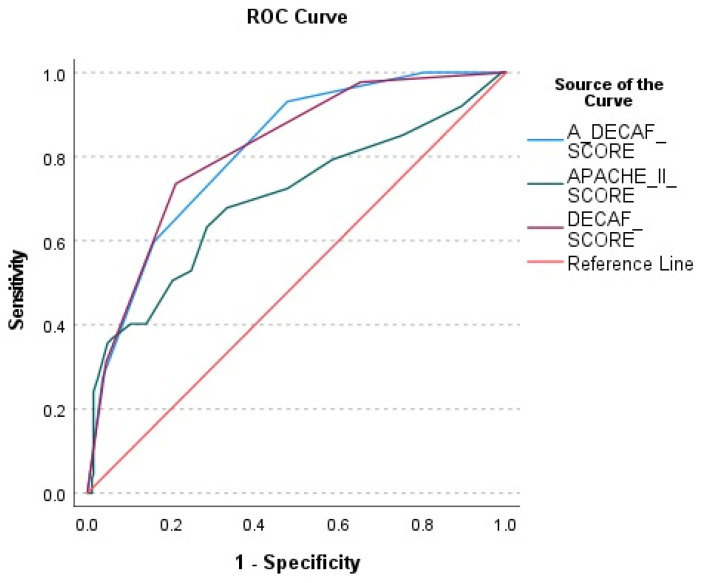
ROC analysis of A-DECAF, DECAF, and APACHE-II scores in predicting patient survival.

The performance of the A-DECAF, DECAF, and APACHE-II scores in predicting patient survival was evaluated using ROC analysis (Figure 2). According to ROC curve analysis, the A-DECAF score demonstrated the best performance in survival prediction, offering higher sensitivity and specificity compared to those of the other scores.

In long-term survival analysis, age and the DECAF score were statistically shown to be significant prognostic factors for survival. To create a more effective prognostic model by combining these two strong variables, the A-DECAF score was developed. By integrating the individual effects of age and the DECAF score, the goal was to achieve greater accuracy in predicting long-term survival.

The performances of APACHE-II, DECAF, and A-DECAF scores in predicting survival were compared using ROC analysis and are presented in Table 3. It was observed that the DECAF (AUC = 0.816) and A-DECAF (AUC = 0.813) scores provided higher accuracy compared to that of the APACHE-II score (AUC = 0.699). The A-DECAF score demonstrated the highest sensitivity (93.1%) in determining survival.

The survival-associated factors were evaluated using univariate and multivariate Cox regression analyses (Table 4). In univariate Cox regression analysis, pH, pCO_2_, age, the presence of lung cancer, hypertension, coronary artery disease, atrial fibrillation, the A-DECAF score, and the need for invasive mechanical ventilation (IMV) were identified as factors associated with survival (*p* < 0.05). In multivariate analysis, lung cancer (HR: 1.922, 95% CI: 1.018–3.630, *p* = 0.044) and the need for IMV (HR: 1.728, 95% CI: 1.028–2.905, *p* = 0.039) were determined as independent predictors of survival. The A-DECAF score demonstrated the strongest association with survival (HR: 2.239, 95% CI: 1.771–2.831, *p* < 0.001), with each unit increase in the score decreasing the chance of survival by approximately 2.2 times.

**Figure 3:**
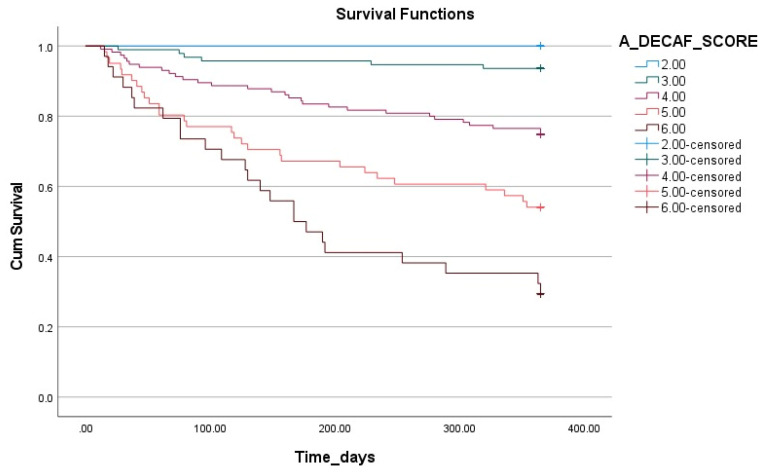
Kaplan–Meier survival analysis based on A-DECAF score.

The impact of the A-DECAF score on survival is illustrated in Figure 3 using Kaplan–Meier survival analysis. This analysis demonstrates that the survival rates significantly decrease as the A-DECAF score increases. The patients with low scores had longer survival durations and higher survival rates, whereas those with high scores (particularly five and six) showed a rapid decline in survival rates in the early period.

## 4. Discussion

This study was conducted to evaluate the prognostic factors influencing long-term survival in patients with persistent HRF secondary to COPD who were followed in an ICU and to analyze the effectiveness of a novel scoring system, the A-DECAF score. The findings of this study indicate that age, pH, pCO_2_, the need for IMV, the presence of lung cancer, and the A-DECAF score have significant impacts on survival. In Cox regression analysis, the A-DECAF score was identified as the strongest independent predictor of survival, while the presence of lung cancer and the need for IMV were also determined as independent factors negatively affecting survival. ROC analysis demonstrated that the A-DECAF score had the highest sensitivity compared to the other scoring systems, showing a superior performance in survival prediction. Kaplan–Meier survival analysis further supported these findings, revealing significantly lower survival rates in the patients with higher A-DECAF scores. This study highlights the potential of the A-DECAF score as a prognostic tool that can be integrated into clinical practice and used as a guide in patient management. However, the validation of this scoring system in different patient populations and larger cohorts is necessary. Such validation studies could contribute to the recognition of the A-DECAF score as a universal prognostic tool.

In recent years, advancements in critical care medicine have enabled not only the management of acute treatment processes, but also the evaluation of long-term outcomes [6]. In this context, post-ICU survival has emerged as a key metric for analyzing the long-term outcomes of those with chronic and critical illnesses [7]. Although numerous studies in the literature focus on HRF-related mortality and the effects of NIMV applications, the data on the long-term prognosis of this patient group remain limited. A recent study that thoroughly examined the underlying causes of HRF and their impact on survival reported a one-year survival rate of 81% [8]. The same study emphasized that the underlying causes of HRF could significantly affect survival; patients with diagnoses such as neuromuscular disease and congestive heart failure had the worst prognosis, whereas patients with HRF caused by factors such as opioid use showed the best outcomes. In our study, the one-year survival rate was calculated to be 75.6%. In our study, the exclusion of etiological factors, such as procedures requiring general anesthesia and/or sedation, as well as opioid use—both of which could contribute to HRF and are associated with a better prognosis—may have contributed to the lower survival rate observed.

The aging population poses significant challenges for healthcare systems [9]. This is reflected in the increasing admission rates of elderly patients to hospitals and ICUs [10]. In a study conducted by Guillon et al. on patients aged ≥80 years, a significant increase in mortality was reported in this population following ICU admission [11]. Similarly, in a study by Barisich et al. examining overall survival after ICU discharge, age was highlighted as an independent risk factor [12]. In our study, age was also identified as a significant prognostic factor for long-term survival. This finding is associated with the negative impact of aging-related comorbidities and reduced physical reserves on the progression of critical illnesses, leading to lower survival rates. Therefore, age should be considered in clinical decision-making processes, and more tailored treatment and follow-up strategies should be developed for elderly individuals.

It is well established that comorbidities have a significant impact on the clinical course, complications, and patient outcomes in the ICU [13]. A large, multicenter observational study by Simpson et al. demonstrated the substantial effects of chronic conditions on ICU management and long-term survival outcomes [14]. Similarly, a study conducted by Hong et al. reported that patients with cancer admitted to the ICU had significantly lower long-term survival rates compared to those of the general critically ill population [15]. In our study, when evaluating the comorbidities that may influence survival in patients with COPD, the presence of lung cancer was identified as a major factor negatively affecting survival. Cox regression analysis further confirmed lung cancer as an independent prognostic factor. These findings underscore the need for the careful assessment of lung cancer in patients with COPD regarding long-term survival. However, while hypertension, coronary artery disease, and atrial fibrillation were found to be associated with survival in univariate analysis, they were not identified as independent predictors in multivariate analysis. This suggests that the impact of these conditions on COPD-related mortality may be influenced by other variables, with lung cancer being a more dominant prognostic factor. Screening for lung cancer and a multidisciplinary follow-up approach may play a crucial role in improving the survival outcomes in patients with COPD.

The DECAF score was developed to predict disease severity and in-hospital mortality in AE-COPD [16]. Patients with high DECAF scores may have increased ICU requirements and require closer monitoring [17]. A recent study also indicated its association with readmissions within 90 days [18]. Furthermore, a study conducted on patients with AE-COPD monitored in the ICU demonstrated the superiority of the DECAF score over other scoring systems in predicting mortality [19]. Considering this information, in this study, long-term survival was evaluated in patients followed up in an ICU with DECAF, AE-COPD, and HRF. The findings showed that the DECAF score outperformed the other scoring systems in predicting long-term survival in the patients with AE-COPD. The DECAF score stands out as a reliable tool in determining not only short-term, but also long-term prognoses, as it includes basic clinical parameters affecting mortality and survival. The ROC analysis performed in this study supported the accuracy of the DECAF score in predicting long-term survival and demonstrated that it provides higher prognostic accuracy compared to the other commonly used systems. Additionally, the A-DECAF score, developed by incorporating the significant effect of age on prognosis, further enhanced the performance of the DECAF score. According to the analysis results, the A-DECAF score demonstrated the best performance in long-term survival prediction, achieving the highest sensitivity. Cox regression analysis revealed that each unit increase in the A-DECAF score was associated with an approximately 2.2-fold reduction in the chance of survival. Kaplan–Meier survival analysis further supported these findings, showing significantly lower survival rates in the patients with higher A-DECAF scores.

These results indicate that the A-DECAF score is a strong prognostic predictor for survival in patients with persistent HRF due to AECOPD and may serve as a valuable tool for patient risk assessment in clinical practice.

The success of the A-DECAF score in predicting long-term survival stems from its ability to provide more comprehensive assessment by incorporating age into the prognostic model. In elderly patients, the burden of comorbidities and diminished physiological reserves increases the risk of mortality during critical illness. Our study demonstrates that the A-DECAF score is a powerful tool for predicting survival rates, particularly in older patient populations. Additionally, it is believed that this scoring system could facilitate personalized approaches in patient management. However, further investigation into the validity of the A-DECAF score in different patient populations, along with comparative analyses with other scoring systems in larger-scale studies, is essential to strengthen its utility in clinical practice. Such studies could contribute not only to AE-COPD treatment, but also to the development of more effective treatment and monitoring strategies for patient groups experiencing HRF due to various underlying causes.

## 5. Limitations

Our study has some limitations. The retrospective design may pose challenges in ensuring the completeness of the data. Additionally, the fact that this research was conducted at a single center is a significant factor limiting the generalizability of the findings. The exclusion of factors associated with a favorable prognosis such as opioid use restricted the ability to fully evaluate their impact on survival rates. Furthermore, the lack of detailed analysis regarding the severity of the included comorbidities made it difficult to fully assess their impact on long-term survival.

## 6. Conclusions

This study evaluated the prognostic factors affecting long-term survival in patients who developed persistent HRF due to COPD. The findings of this study indicate that the A-DECAF score is the strongest independent predictor of survival, while the presence of lung cancer and the need for IMV were also identified as independent factors negatively affecting survival. The incorporation of age into the DECAF score to form the A-DECAF score resulted in the highest sensitivity and accuracy for long-term survival prediction, demonstrating a superior performance compared to those of the existing scoring systems. Particularly in elderly patients, the A-DECAF score emerges as a reliable prognostic tool that can guide clinical decision making. These results suggest that integrating the A-DECAF score into clinical practice may significantly contribute to patient management. However, further validation in different patient populations and larger cohorts is necessary to establish its generalizability and clinical applicability.

## Figures and Tables

**Table 1:** Characteristics of patients based on survival status.

Characteristics	Deceased Patients (N = 87) (24.4%)	Surviving Patients (N = 270) (75.6%)	*p*-Value
Age, years (Mean ± SD)	73.21 ± 10.92	67.41 ± 11.15	<0.001
Male Gender	60 (69%)	166 (61.5%)	0.208
Long-term oxygen therapy due to chronic respiratory failure	67 (64.4%)	172 (68%)	0.516
**Comorbidities**			
Emphysema	20 (23.8%)	96 (37.2%)	0.024
Lung Cancer	15 (17.2%)	7 (2.6%)	<0.001
Hypertension	56 (64.4%)	136 (50.4%)	0.023
Coronary Artery Disease	23 (26.4%)	41 (15.2%)	0.017
Atrial Fibrillation	19 (21.8%)	29 (10.7%)	0.008
APACHE-II Score	23 ± 5	19 ± 4	<0.001
Need for Invasive Mechanical Ventilation	21 (24.7%)	28 (10.5%)	<0.001
DECAF Score	4.02 ± 0.80	2.91 ± 0.82	<0.001
A-DECAF Score	4.80 ± 0.92	3.47 ± 1.05	<0.001

**Table 2:** Evaluation of patients’ initial blood test results.

Laboratory Findings	All Patients (N = 357) Median (Interquartile Range (25–75))	Deceased Patients (N = 87) Median (Interquartile Range (25–75))	Surviving Patients (N = 270) Median (Interquartile Range (25–75))	*p*-Value
Blood Urea Nitrogen	50 (33–72)	56 (38–89)	47 (31–66)	0.001
Creatinine	1 (0.80–1.28)	1 (0.79–1.35)	1 (0.80–1.26)	0.940
pH	7.31 (7.24–7.38)	7.29 (7.22–7.33)	7.33 (7.25–7.39)	<0.001
pCO_2_	72 (65–86)	77 (68–88)	71 (64–84)	0.003

**Table 3:** ROC analysis results for APACHE-II, DECAF, and A-DECAF scores in predicting survival.

Variable	AUC	95% Confidence Interval	Cut-Off Value	Sensitivity (%)	Specificity (%)	PPV (%)	NPV (%)	LR+	LR−	*p*-Value
APACHE-II	0.699	0.629–0.768	20.50	63.2	71.5	41.7	85.8	2.22	0.51	<0.001
DECAF Score	0.816	0.766–0.865	3.50	73.6	78.9	52.9	90.3	3.48	0.34	<0.001
A-DECAF Score	0.813	0.765–0.861	3.50	93.1	52.2	38.6	95.9	1.95	0.13	<0.001

**Table 4:** Cox regression analysis results of factors associated with survival.

Variable	Univariate Cox Regression		Multivariate Cox Regression	
	HR (95% CI)	*p*-Value	HR (95% CI)	*p*-Value
pH	0.037 (0.005–0.277)	<0.001	-	-
pCO_2_	1.014 (1.003–1.024)	0.009	-	-
Age	1.041 (1.022–1.060)	<0.001	-	-
Lung cancer	4.916 (2.811–8.598)	<0.001	1.922 (1.018–3.630)	0.044
Hypertension	1.604 (1.034–2.488)	0.035	1.157 (0.728–1.837)	0.537
Coronary artery disease	1.700 (1.055–2.7348)	0.029	1.339 (0.817–2.194)	0.247
Atrial fibrillation	1.995 (1.199–3.319)	0.008	0.790 (0.443–1.409)	0.424
A-DECAF Score	2.458 (2.017–2.995)	<0.001	2.239 (1.771–2.831)	<0.001
Need for IMV *	2.481 (1.515–4.063)	<0.001	1.728 (1.028–2.905)	0.039

* IMV: Invasive mechanical ventilation.

## Data Availability

The original contributions presented in this study are included in this article. Further inquiries can be directed to the corresponding author.
